# Investigation of the Effect of Contact Seal Geometry on Frictional Moment in Ball Bearings

**DOI:** 10.3390/ma18225068

**Published:** 2025-11-07

**Authors:** Paweł Zmarzły, Mateusz Wrzochal, Anna Rębosz-Kurdek

**Affiliations:** 1 Faculty of Mechatronics and Mechanical Engineering, Kielce University of Technology, al. Tysiąclecia Państwa Polskiego 7, 25-314 Kielce, Poland; 2 Institute of Manufacturing and Materials Technology, Gdańsk University of Technology, Gabriela Narutowicza 11/12, 80-222 Gdańsk, Poland; mateusz.wrzochal@pg.edu.pl; 3 Faculty of Management and Computer Modelling, Kielce University of Technology, al. Tysiąclecia Państwa Polskiego 7, 25-314 Kielce, Poland; arebosz@tu.kielce.pl

**Keywords:** rolling bearings, contact seals, frictional moment, roundness, waviness

## Abstract

Due to their simple design and versatility, rolling bearings are widely used in various industrial and engineering applications. One of the key parameters characterizing ball bearings is the frictional moment (also referred to as resisting torque). Excessive torque values can increase energy consumption, which is undesirable from an energy efficiency standpoint. In response to the increasing demand for energy-efficient solutions, studies on the frictional moments of ball bearings are gaining particular significance. Numerous research studies have been conducted to investigate the factors that affect this parameter in rolling bearings. However, in the case of rolling bearings with contact seals, accurately evaluating these relationships is challenging due to the instability of frictional moment values observed during measurements. Therefore, this paper presents a study aimed at evaluating the impact of rubber seal geometry (specifically roundness and waviness deviations) on the value of friction torque in 6304-type ball bearings. It is important to note that manufacturers employ various types of seals. This study presents a preliminary qualitative assessments of the manufacturing quality of rubber contact seals from selected producers. Form deviations of the rubber seals were analyzed using a multisensor O-Inspect coordinate measuring machine. The frictional moment of rolling bearings was measured using a dedicated measurement system developed at Kielce University of Technology. Measurements were conducted under two axial load values (70 N and 135 N) and two rotational speeds (50 rpm and 1800 rpm). Based on qualitative observations, the dominant factor influencing the frictional moment magnitude was identified.

## 1. Introduction

Rolling ball bearings are manufactured with two sealing options: open bearings (without seals) and closed bearings (with seals). Although open-type rolling bearings are less expensive to produce and have lower frictional moment values, they are increasingly less used in practice due to their need for external lubrication. Additionally, bearings without seals are exposed to external contaminants, which significantly shortens their lifespan. As a result, sealed-type bearings with factory-installed seals are now more commonly used. The use of seals in bearings reduces the risk of contaminants and moisture penetrating the interior, which can cause internal damage to bearing components (balls, raceways) and lead to corrosion. Furthermore, the sealing of rolling bearings prevents the lubricant from leaking out of the bearing and migrating to the mechanisms in which the bearing is installed. Rolling bearing seals can be classified into four main types: contact seals, non-contact seals, static seals, and special seals.

Contact seals provide high protection effectiveness against external contaminants. In this type of seal, the sealing lips are in contact with the inner ring, which may lead to increased frictional moment. Due to the higher friction, bearings with contact seals operate at lower rotational speeds. Bearings with non-contact seals typically use metal sealing plates (ZZ-type bearings) or molded rubber seals (2RU-type bearings), which do not make contact with the inner ring. Compared to bearings with contact seals, they exhibit lower frictional moment and can operate at higher rotational speeds. However, their sealing performance is lower, so they should be used in less dusty and less aggressive environments. In the case of static seal bearings, the sealing elements are placed between fixed components of the bearing. This solution is typically used in bearings operating at low rotational speeds, where minimizing friction and ensuring stable frictional moment are particularly important. Static bearings also offer greater resistance to certain types of mechanical loads, but their proper functioning requires precise assembly. Bearings with special seals include, among others, bearings with labyrinth seals, in which grease fills the labyrinth, creating a physical barrier against contaminants. Another example of a special seal is the felt seal, which uses oil-impregnated felt. Bearings with special seals are characterized by high effectiveness in protecting against contamination, and can operate in more demanding environmental conditions and at higher speeds, but their production is more complex and operating costs are higher.

A review of the literature indicates a limited number of scientific publications concerning the study of seals in ball rolling bearings. In the study [1], the authors focus on the problem of sealing failure in double-lip seal rings used in high-speed ball bearings for unmanned aerial vehicles. The research results indicate that the contact pressure at the main lip of the seal was the highest. In study [2], experimental and numerical investigations were conducted to examine the influence of seal contact force in ball bearings on the value of the frictional moment. Additionally, the dimensional accuracy of the seals was assessed using an optical measurement system. It was demonstrated that an increase in seal stiffness leads to a rise in frictional moment and bearing operating temperature, which is an undesirable phenomenon. Similar research is presented in article [3], where the influence of the inclination angle of the sealing lip in high-speed micro bearings on the friction coefficient was evaluated. The paper [4] presents the effect of symmetric and asymmetric semi-metallic gasket core shapes on the sealing performance in bolted flange joints. Publication [5] presents research results on the influence of lubricating film thickness on the operating characteristics of ball bearings. Study [6] includes both simulation and experimental investigations, focusing, among other things, on the effect of the sealing lip inclination angle on frictional moment in ball bearings. Paper [7] presents research on grease durability depending on selected sealing parameters. In paper [8], a comprehensive review of factors affecting energy consumption in rolling bearings is presented, highlighting that frictional moment of seals plays a significant role in determining the energy efficiency of these mechanisms. Interesting research is presented in article [9], where computed tomography was used to assess the behavior of grease inside a bearing without the need for disassembly. It is worth noting that computed tomography can be effectively used to evaluate the dimensional and form accuracy of mechanical components, including rolling bearings [10]. Paper [11] presents a numerical analysis of seals in the rotor–bearing–seal system under high-temperature conditions typical for gas turbines. The authors of the study [12] investigated modifications to the geometry of the sealing lip in ball bearings and evaluated its impact on frictional moment and resistance to external contamination. In paper [13], the influence of labyrinth and lip seals on energy consumption in belt conveyor systems was assessed. It was shown that the use of larger tensioning rollers can reduce system efficiency by as much as 40–55%.

In the paper [14], a thermal–stress–wear coupled finite element model of an oil seal was developed, and the coupled dynamic differential equations of the airframe rod end bearing and the oil seal were established. The oil seal parameters were optimized, and test results showed that the average grease retention rate of the optimized oil seal increased by 17.3%. Huang and Li [15] presented an experimental study and simulation analysis on the seal failure of the cone bit bearing seal. The wear rate of the new structure was reduced to 20–38% of that of the original structure. The paper [16] proposes a novel ceramic-coated floating oil seal (NCCFOS) composite structure that enhances wear resistance without modifying the existing sealing cavity configuration.

One of the fundamental operational parameters of ball rolling bearings is their frictional moment [13,17]. Currently, efforts are being made to produce bearings characterized by low values of this parameter, as excessive frictional moment leads to increased energy consumption by mechanical systems, which is undesirable [18]. This is particularly important in the components of electric and hybrid vehicle drivetrains, where energy losses need to be minimized [19]. The value of frictional moment is influenced by many factors, such as the geometry of the inner and outer rings, radial clearance, type of grease used, bearing load, and rotational speed [20,21]. However, a significant factor is the type and geometry of the seal used [2]. Even when a specific type of seal is applied, the quality of its manufacture significantly affects the bearing’s tightness and, consequently, its performance parameters. Therefore, it is necessary to thoroughly examine the dimensional and form accuracy of seals. It is also worth noting that rolling bearing manufacturers, despite using the same type of bearings and seals, apply different geometries of sealing lips and the seals themselves. In many cases, seal quality assessment is limited to measuring external and internal dimensions, which often proves insufficient, as it does not allow for the detection of detailed defects and deformations. Expanding the research to include the evaluation of roundness deviations of seals enables the identification of defects such as ovality, which directly affect sealing quality and operational parameters, including frictional moment. It should be emphasized that measurements of roundness and waviness deviations are commonly used to assess the manufacturing quality of bearing rings [21], whereas the evaluation of seal geometry using these parameters is still rarely applied.

This article presents preliminary research aimed at evaluating the impact of roundness and waviness deviations of the bearing rings in type 6304 2RS bearings on the value of the frictional moment. Bearings of the same type from five different manufacturers were selected for analysis. The study also considered additional parameters such as rotational speed and axial load.

Due to the limited number of samples, the obtained results are qualitative and preliminary. They may serve as a starting point for a more extensive analysis once a larger dataset is collected. Despite the preliminary nature of the research, the results provide valuable insights for designers and manufacturers of rolling bearings, supporting the development of products with low frictional moment. Such an effect can contribute to reduced energy consumption by mechanical systems, which is particularly important in applications requiring high energy efficiency.

## 2. Materials and Methods

The research presented in this article was divided into two main stages. In the first stage, the frictional moment was measured using a test rig developed at the Kielce University of Technology (Kielce, Poland). After measuring the frictional moment, the rolling bearings were disassembled to gain access to the sealing rings. Subsequently, after cleaning the seals of grease residues, roundness and waviness profiles were measured using the Zeiss O-Inspect optical vision system.

### 2.1. Rolling Bearing Seals

**Figure 1:**
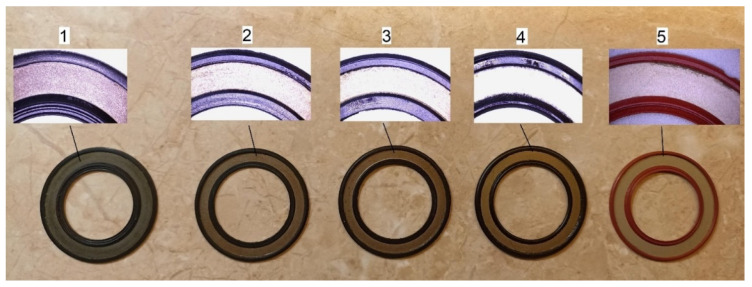
Examined Rolling Bearing Seals.

Rolling bearings of the type 6304 2RS were selected for the study. These are single-row ball bearings, sealed on both sides with contact rubber seals. It should be noted that bearing manufacturers, despite using the same type designation, apply seals with varying lip geometries, which are typically considered company trade secrets. Therefore, bearings of the same type from five different manufacturers, labeled with numbers 1–5, were used for the analysis. The bearings were made of AISI 52100 bearing steel. Figure 1 shows photographs of the applied seals along with magnified views obtained using an optical microscope.

### 2.2. Frictional Moment Measurement

**Figure 2:**
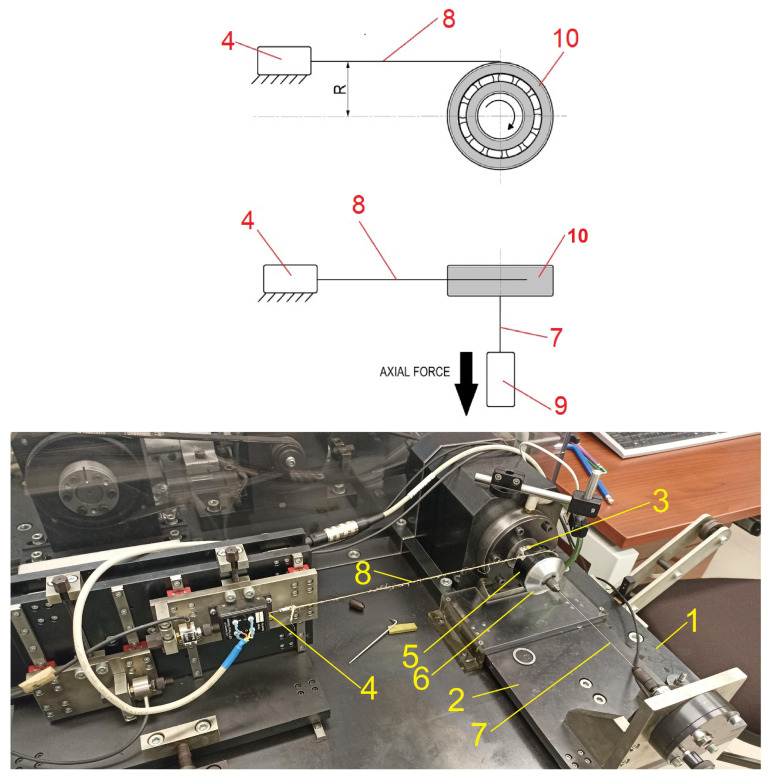
Device for measuring the frictional moment of ball bearings.

Frictional moment is an important operational parameter of ball bearings, as it determines the energy efficiency of bearing assemblies. It can be defined as the moment of force that must be overcome for the bearing to rotate. The total friction generated within the bearing results from a complex interaction of various physical and structural mechanisms. It consists of rolling and sliding friction occurring in the contact zone between rolling elements and raceways, on the contact surfaces between rolling elements and the cage, as well as on the guiding surfaces of the rolling elements and the cage. Friction within the grease medium also plays a significant role, and its characteristics depend on viscosity, temperature, and the dynamics of grease flow [2,22]. In the case of contact-sealed bearings using rubber seals of the “RS” type, sliding friction occurring on the contact surfaces between the seal and the inner ring has a dominant influence on the value of the frictional moment. Therefore, the geometry of the seals should be thoroughly analyzed in the context of the frictional moment of rolling bearings. A specially designed measuring device, shown in Figure 2, was used to study the frictional moment.

On the main base (1) of the measuring device, a longitudinal load table (2) is mounted, which cooperates with an actuator fixed to the underside of the base. The table is integrated with an axial force sensor. On the left side of the spindle (3), a table is placed to which force gauges (4) can be connected. The drive unit provides smooth, adjustable spindle rotation speeds up to 26,000 rpm. The outer ring of the tested bearing is placed in a thin-walled housing made of aluminum alloy (5), with a flange that prevents the bearing from falling out on the opposite side. The tested bearing (10) is mounted on a shaft with a sliding fit and is fixed by the face of the inner ring using a screw threaded into the shaft via a washer. A cover (6) is screwed onto the housing. At the center of the cover, a rod is attached to apply axial load (7). A rod mounting system is located on the surface forming the bearing housing. One force gauge (9) is mounted on the measuring table and connected to the measuring housing via a rod (8). Frictional moment occurring in the bearing causes the outer ring and housing to attempt to rotate, which transfers force through the rod proportional to the resisting moment. The software then records the resulting changes and, based on the input data of the tested bearing, calculates the resisting moment. The presented test rig performs frictional moment measurements at a frequency of every 0.02 s. The rig allows for adjustment of the bearing’s rotational speed as well as the values of applied axial and radial loads.

At the beginning of the test, the rolling bearing, enclosed in a housing and prepared for measurement, was loaded with a given axial force (70 N or 150 N, depending on the test variant), and then the inner ring was set in rotation by means of the spindle shaft at a speed of 50 rpm. For such a low rotational speed, the resistance torque was, in most cases, low and stable (the observed fluctuations of the measurement result over time were small), the temperature practically did not increase, and therefore the trend of the frictional moment over a longer period of time had an approximately constant trend. After 30 s of operation with such measurement parameters, the first result was recorded, and then the rotational speed was increased to 1800 rpm. For such conditions, the frictional moment was often unstable (significant fluctuations over time), and a decreasing trend was observed. The decrease in the frictional moment value over time is caused by the increase in the bearing temperature, which in turn is caused by more intense friction. The systematically decreasing viscosity of the grease causes the generation of lower resistance to motion. The test was carried out until the bearing frictional moment was relatively stable, i.e., approximately 60 s after the speed jump. Then the second measurement result was recorded. Then the bearing was stopped, the axial load was removed, and then the bearing was removed from the housing. The system was cooled to ensure the same initial conditions each time. Then another bearing was used for another test. This measurement methodology ensured that the resistance torque results were obtained under two different operating conditions: low rotational speed, observed temperature increase, and stable operation; and high rotational speed, observed temperature increase, and less stable bearing operation over time.

### 2.3. Roundness and Waviness Measurement

In many cases, the assessment of the manufacturing quality of cylindrical-shaped components is based exclusively on diameter measurement. This is a significant limitation, as it does not provide information about the nature of surface profile irregularities on the cylindrical surface. For critical components, such as rolling bearing parts, it is therefore necessary to extend the inspection to include an analysis of geometric deviations. One of the key deviations is roundness deviation, defined as the largest difference between the actual surface profile and the ideal circle best fitted to that profile. The smaller the roundness deviation, the more the measured profile resembles a perfect circle. A variety of methods are used to assess roundness deviation, which can generally be classified into methods based on rotational datum method and reference methods, such as the V-block method. Rotational datum method offer the highest accuracy but are mainly used for measurements in laboratory conditions or industrial metrology laboratories. In recent years, we have observed rapid development in coordinate measuring technology. Modern coordinate measuring machines (CMMs) allow not only precise dimensional measurements but also satisfactory evaluation of form deviations. Traditionally, these machines are equipped with contact probes, but nowadays multi-sensor CMMs are also available, which, in addition to contact probes, include optical systems enabling non-contact surface measurement [23,24]. It should be noted that contact measurement of form deviations in components made of elastic materials, such as rubber seals, is not recommended, as the contact from the measuring probe may cause elastic deformation of the tested material, resulting in distorted measurement results. In such cases, optical measurement methods are advised.

**Figure 3:**
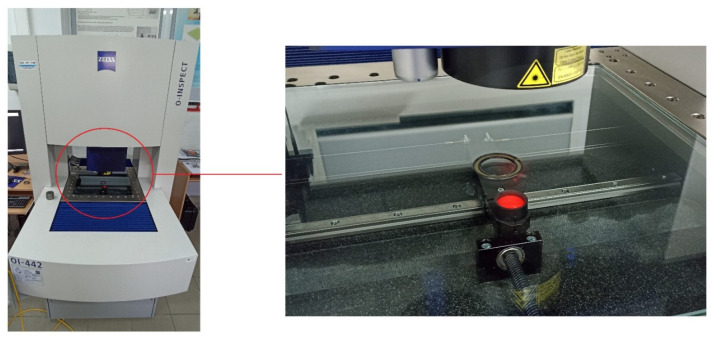
Roundness and waviness measurement.

To perform the measurement of roundness and waviness deviations, a multisensor coordinate measuring machine, O-Inspect by Zeiss (Oberkochen, Germany), was used. The measurement was carried out using an optical method with an optical system magnification of 0.5X and using transmitted light. The measured profile was then filtered using a Gaussian filter in two ranges: 2–15 undulations per revolution (for roundness) and 15–500 undulations per revolution (for waviness). Based on the reference circle determined using the least squares method (LS mean circle), the roundness deviation RONt_2–15_ and waviness deviation RONt_15–500_ were calculated. Each ring was measured ten times. Table 1 presents the average values from 10 roundness and waviness deviation measurements. Figure 3 shows the measurement of roundness and waviness deviations of sealing rings using O-Inspect multisensor coordinate measuring machine.

## 3. Results and Discussion

To conduct a detailed analysis of the influence of selected factors on the value of the frictional moment, the test results were presented in Table 1. The main objective of the study was to evaluate the impact of roundness and waviness deviations of the sealing lip on the frictional moment of rolling bearings, taking into account two rotational speeds and axial loads. Two bearings from each manufacturer, labeled with numbers from 1 to 5, were tested.

Analyzing the results presented in Table 1, it can be observed that the frictional moment varies among bearings of the equivalent type provided by various producers. Moreover, the frictional moment values for bearings from the same manufacturer showed certain discrepancies (see bearings 4a and 4b in Table 1), which may result from an improperly conducted manufacturing process. Based on the analysis of the operational parameters of the tested rolling bearings, it can be clearly stated that an increase in rotational speed leads to an increase in frictional moment. This effect is particularly noticeable at lower axial load values. However, when the axial load is increased from 70 N to 135 N, a slight decrease in frictional moment is observed, which may result from seal deformation caused by high axial load.

When evaluating the roundness and waviness deviations of the sealing lip in RS-type seals, significant disparities between products offered by different manufacturers are once again observed. However, it is worth noting that the differences in roundness deviations between seals from the same manufacturer are not as pronounced as in the case of frictional moment. A comprehensive analysis of form deviations revealed that the highest values of roundness and waviness deviations were observed for bearing no. 3a. Visual inspection of seal images obtained using an optical microscope (see Figure 1) shows numerous individual grooves on the sealing lip of bearing no. 3. In contrast, the edges of the seals in bearings no. 1 and 5 exhibit a more regular shape, which is reflected in lower roundness and waviness deviation values. An analysis of the influence of roundness deviation RONt_2–15_ on the frictional moment value indicated that bearings with sealing rings exhibiting higher roundness deviations generate lower frictional moment. It is important to note that rings with lower roundness deviation have a shape closer to a perfect circle, resulting in better contact with the inner ring and more effective sealing. However, a larger contact area between the seal and the inner ring leads to an increase in the frictional moment. A similar relationship is observed in the case of waviness deviation in the range of 15–500 undulations per revolution. In this case, waviness has an even stronger impact on the frictional moment, especially at a lower axial load of 70 N. It should also be added that a higher axial load may cause a more uniform distribution of forces around the seal circumference, which can mitigate the influence of seal defects on the frictional moment value.

**Figure 4:**
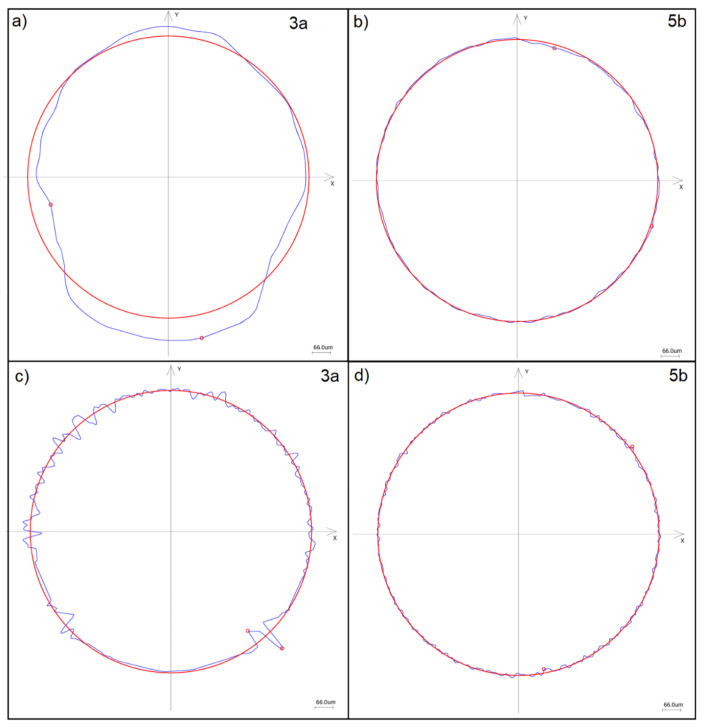
Roundness and waviness profile: (**a**) roundness profile of sealing ring of bearing no. 3a, (**b**) roundness profile of sealing ring of bearing no. 5b, (**c**) waviness profile of sealing ring of bearing no. 3a, (**d**) waviness profile of sealing ring of bearing no. 5b.

To qualitatively assess the nature of irregularities in the sealing lip, Figure 4 presents the roundness and waviness profiles for two selected sealing rings with extreme roundness deviation values (see 3a and 5b in Table 1).

Considering the character of surface irregularities in the seals, it can be stated that the dominant form error in the sealing rings is ovality, which is particularly evident in the case of ring 3a (see Figure 4a). Additionally, when analyzing the waviness profile of the same ring (Figure 4c), numerous individual irregularities can be observed in the upper part of the profile, which may result from residual sprue left over from the sealing ring manufacturing process. This phenomenon is also visible in Figure 1, which presents a microscopic view of the seals. This confirms that higher values of roundness and waviness deviations reduce the contact surface between the sealing lip and the inner ring, resulting in a lower frictional moment. On the other hand, when analyzing the roundness and waviness profiles of the sealing ring with the lowest deviation values (Figure 4b,d), we can see that the measured profiles are closer to the nominal circle, indicating higher seal quality.

To ensure more comprehensive interpretation of the measurement data, a correlation analysis was conducted between the frictional moment (all 10 bearings). Coefficients of determination (R^2^) were calculated to quantify the strength of these relationships. The results were visualized using scatter plots, incorporating two key variables for frictional moment measurements: rotational speed (50 rpm and 1800 rpm) and axial load (70 N and 135 N).

**Figure 5:**
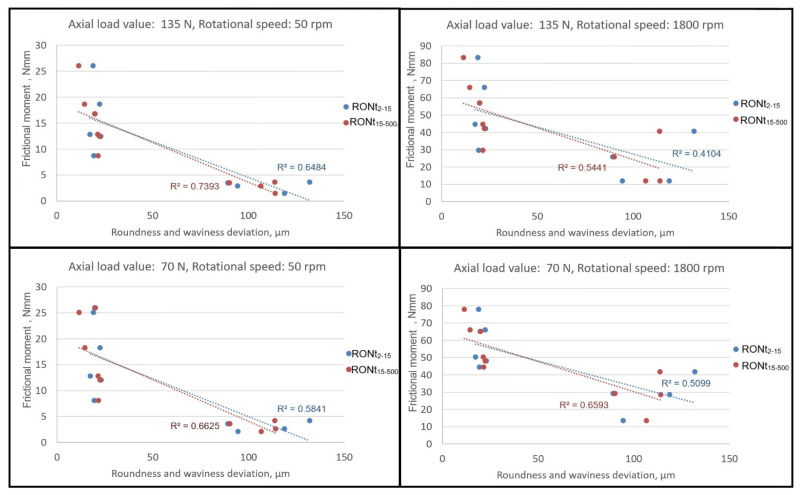
Impact of the roundness and waviness deviation of the 6304 2RS bearing seal on the frictional moment values.

Figure 5 shows dependence of the frictional moment value on the roundness and waviness deviation values.

Preliminary analysis of the results indicates that higher roundness and waviness deviations of the sealing rings may be associated with a reduction in resistance torque. This trend was observed in the tested samples across both rotational speeds (50 rpm and 1800 rpm) and axial loads (70 N and 135 N). Preliminary coefficients of determination (R^2^) suggest a potentially stronger association between resistance torque and waviness deviation compared to roundness deviation. These observations are indicative rather than conclusive due to the limited sample size.

## 4. Conclusions

Increasing demands for energy efficiency in mechanical devices, particularly in the electromobility sector, are driving the use of rolling bearings that generate lower frictional moment values. As a result, there is a need to identify the factors that predominantly influence the frictional moment and to implement solutions that allow its reduction. It is worth noting that manufacturers use seals with varied geometries in their products, which are typically considered trade secrets.

Measuring a dynamic parameter such as frictional moment in rolling bearings is subject to numerous random and systematic errors. These errors arise from both the measurement conditions and the inherent characteristics of the bearing itself. Temperature plays a significant role in influencing frictional moment: as temperature increases, lubricant viscosity decreases, reducing viscous friction and thereby lowering overall friction torque. Simultaneously, thermal expansion of bearing components can alter internal clearances, and in cases of overheating, may lead to increased mechanical friction. Geometric deviations in the raceways or rolling elements can result in uneven pressure distribution within contact zones, leading to localized friction increases, vibration, and unstable torque readings. In precision bearings, even small geometric imperfections can significantly impact measurement repeatability.

The results of the study presented in the article revealed discrepancies in the frictional moment values of bearings of the same type offered by different manufacturers. It was demonstrated that seal form errors have a significant impact on the frictional moment value. Bearings with seals exhibiting greater roundness and waviness deviations generate lower frictional moment, which may result from incomplete contact between the seals and the bearing rings, leading to reduced friction force. This effect is particularly noticeable in the case of waviness analyzed in the range of 15–500 undulations per revolution. Additionally, to accurately assess the geometry of rubber seals, vision-based measurement systems can be successfully used, enabling a better correlation between geometric parameters and frictional moment values.

The research presented in the article should be considered preliminary and qualitative, aimed at evaluating the influence of seal geometry features on the frictional moment value. Due to the limited amount of data, the obtained results should be interpreted with caution, as they do not yet provide a basis for comprehensive quantitative analysis. However, they indicate that roundness and waviness deviations of the sealing lip can be treated as important criteria for assessing the manufacturing quality of sealing rings in ball bearings.

In future research, after collecting a larger number of samples, correlation and regression analyses are planned. These will allow for a quantitative determination of the strength and significance of the influence of individual geometric features on the frictional moment, and for the development of predictive models. Based on this, it will be possible to extend the research to formulating and solving the problem of optimal seal design. The objective of this problem is to minimize the frictional moment, taking into account geometric, technological, and quality constraints. The authors will focus on examining the surface topography of seals in the context of assessing their contact with the working surfaces of rolling bearings. In addition, the authors use computed tomography to assess the contact surface of seals with bearing rings.

## Figures and Tables

**Table 1:** Research results.

Bearing Number	Frictional Moment Values, Nmm				Roundness and Waviness Deviation, µm	
	Axial Load Value: 135 N		Axial Load Value: 70 N		RONt_2–15_	RONt_15–500_
	Rotational Speed: 50 rpm	Rotational Speed: 1800 rpm	Rotational Speed: 50 rpm	Rotational Speed: 1800 rpm		
1a	16.77	56.93	26.01	65.17	19.63	19.985
1b	12.49	42.19	12.07	47.99	22.975	22.38
2a	1.48	12	2.64	28.51	118.79	114.01
2b	2.9	11.98	2.13	13.6	94.375	106.555
3a	3.64	40.58	4.22	41.8	131.92	113.71
3b	3.47	25.8	3.62	29.22	89.235	90.135
4a	26.05	83.19	25.1	77.85	18.94	11.415
4b	18.66	66.04	18.27	66.1	22.33	14.535
5a	8.74	29.73	8.09	44.5	19.35	21.56
5b	12.81	44.77	12.84	50.34	17.385	21.43

## Data Availability

The data presented in this study are available on request from the corresponding author due to privacy.
